# The Role and Mechanism of Oxidative Stress and Nuclear Receptors in the Development of NAFLD

**DOI:** 10.1155/2021/6889533

**Published:** 2021-10-27

**Authors:** Ting Hong, Yiyan Chen, Xiaoying Li, Yan Lu

**Affiliations:** Key Laboratory of Metabolism and Molecular Medicine, Ministry of Education and Department of Endocrinology and Metabolism, Zhongshan Hospital, Fudan University, Shanghai, China

## Abstract

The overproduction of reactive oxygen species (ROS) and consequent oxidative stress contribute to the pathogenesis of acute and chronic liver diseases. It is now acknowledged that nonalcoholic fatty liver disease (NAFLD) is characterized as a redox-centered disease due to the role of ROS in hepatic metabolism. However, the underlying mechanisms accounting for these alternations are not completely understood. Several nuclear receptors (NRs) are dysregulated in NAFLD, and have a direct influence on the expression of a set of genes relating to the progress of hepatic lipid homeostasis and ROS generation. Meanwhile, the NRs act as redox sensors in response to metabolic stress. Therefore, targeting NRs may represent a promising strategy for improving oxidation damage and treating NAFLD. This review summarizes the link between impaired lipid metabolism and oxidative stress and highlights some NRs involved in regulating oxidant/antioxidant turnover in the context of NAFLD, shedding light on potential therapies based on NR-mediated modulation of ROS generation and lipid accumulation.

## 1. Introduction

Nonalcoholic fatty liver disease (NAFLD), a pandemic disease, is predicted to be the most common indication for liver transplantation in the next decade. NAFLD refers to the state when hepatic lipid accounts for more than 5% of the liver weight without excessive alcohol consumption or other known causes of liver diseases (viruses, drugs, toxins, autoimmune disease, etc.). Regarding the clinical course, the full spectrum of NAFLD includes simple steatosis, steatohepatitis, liver cirrhosis, and hepatocellular carcinoma (HCC). Recently, based on the finding of concomitant liver disease and the heterogeneous pathology, a new definition of metabolic dysfunction-associated fatty liver disease (MAFLD) has recently been put forward [1].

According to recent data, the overall global prevalence of NAFLD is estimated to be 24% among adults. The highest prevalence was reported to be 31.79% in the Middle East, followed by 30.45% in South America, 27.37% in Asia, 24.13% in North America, 23.71% in Europe, and 13.48% in Africa [2]. From 2012 to 2017, cirrhosis due to NAFLD and NAFLD-related death increased globally, especially in Australia, Latin America, and Asia [3]. NAFLD has replaced viral hepatitis to be the most common liver disease in China. The prevalence of NAFLD is higher in younger generations and lean people, in addition to the elderly population [4, 5]. Importantly, although there are no typical symptoms or signs of NAFLD, its potential harm to the liver and extrahepatic complications in cardiovascular and other systems cannot be neglected. NAFLD may start with the insulin resistance and dysfunction of adipocytes, providing a pathogenic milieu rich in lipid metabolites, and then, proinflammatory cytokines may be released into the peripheral circulation, leading to mitochondrial dysfunction, activation of apoptosis, and a chronic inflammatory state. The whole process includes crosstalk among the liver, muscles, adipose tissues, and a systemic disturbance of cytokines and hormones, which eventually causes systemic effects such as metabolic syndrome (MetS), type 2 diabetes mellitus (T2DM), cardiovascular disease (CVD), and hypertension. Due to inadequate awareness, unavailability of diagnostic tools, and a lack of effective medication, the vast majority of potential NAFLD patients are undiagnosed and untreated [6].

According to the classical “two-hit” theory, NAFLD is characterized by two steps of liver injury: intrahepatic lipid accumulation (hepatic steatosis) and inflammatory progression to nonalcoholic steatohepatitis (NASH) [7]. This classical hypothesis has been modified to indicate that NAFLD may be a consequence of parallel “multihits” [8]. Lipotoxicity primes the liver for injury arising from “multiple and parallel hits” (oxidative stress and the activation of proinflammatory and fibrogenic pathways) [9]. Oxidative stress leads to cellular dysfunction and is considered a causative factor in the pathophysiology of NAFLD. When the generation of reactive oxygen species (ROS) exceeds the capacity of antioxidants to detoxify them, these highly toxic molecules induce damage to the normal lipid metabolism [10]. Moreover, increased ROS levels are responsible for insulin resistance in numerous settings [11], which indicates that redox-dependent molecular alterations also play an important role in the early stage of NAFLD.

Considering the vital role played by several nuclear receptors (NRs) and transcription factors in the development of NAFLD [12], this review focuses on the role played by ROS in the regulation of the transcriptional network that modulates hepatic lipid metabolism, suggesting a redox-centered pathogenic hypothesis. Moreover, the impact of endogenous hormones as environmental factors on NR expression in the development of NAFLD is discussed.

## 2. ROS Production and Oxidative Stress in the Development of NAFLD

**Figure 1:**
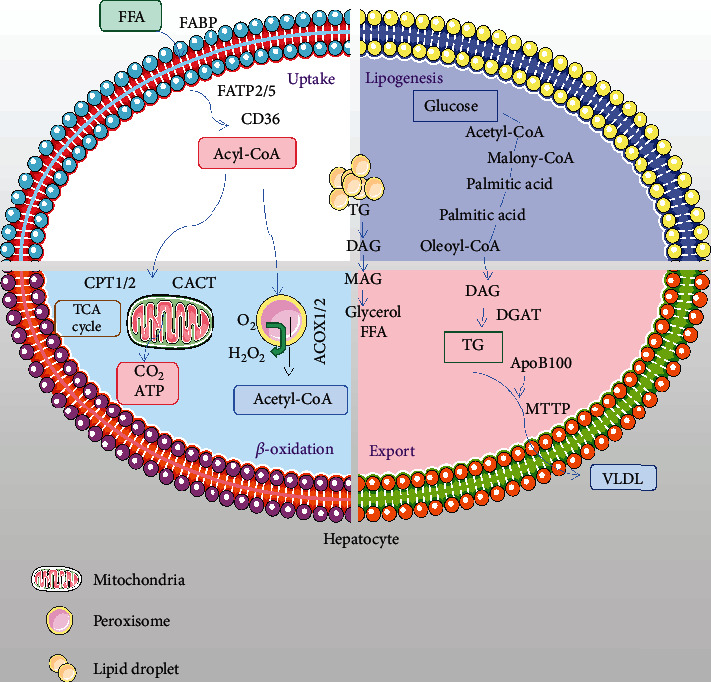
Hepatic lipid metabolism. Increased uptake of circulating free fatty acids (FFA) and *de novo* lipogenesis, impaired oxidation of fatty acids in league with decreased lipids export in the liver all contribute to the development of fatty liver.

The liver serves as the distribution center of nutrients, smoothing out blood glucose and lipid fluctuations between intermittent food intake. The content of triglycerides in the liver varies with the metabolic states. During fasting, fatty acids released from adipose tissues oxidize in hepatic mitochondria to generate energy. On the other hand, when fatty acids and chylomicrons are redundant in the circulation after a meal, the liver packages them in the form of lipid droplets for further use. As shown in Figure 1, increased uptake of free fatty acids and lipogenesis, defects in fatty acids oxidation, and decreased lipids export contribute to the impaired hepatic lipid metabolism. It is worth mentioning that ROS appears necessary in those processes that lead to the dysfunction of lipid metabolism and the development of NAFLD. The imbalance between ROS generation and antioxidant defenses causes oxidative stress and tissue damage [13]. Clinically, increased mitochondrial levels of ROS and mitochondrial dysfunction are observed in liver tissues from patients with NAFLD [14, 15]. The results from mouse models also indicate that impaired mitochondrial dynamics leads to metabolic abnormalities such as NASH phenotypes [16]. This section outlines the knowledge on ROS generation and highlights the role of oxidative stress in the NAFLD pathology.

### 2.1. Mechanism of Excessive ROS Production in NAFLD

ROS or oxidants can be classified as free radicals and major physiologically relevant ROS, including superoxide anions (O_2_^•−^), hydroxyl radicals (•OH), and hydrogen peroxide (H_2_O_2_). The imbalance between oxidants and antioxidants induces the oxidative stress [17]. Under conditions of normal antioxidant homeostasis, cells can effectively remove physiological ROS through protection systems consisting of enzymatic and nonenzymatic components. Some of the most relevant enzymes that detoxify ROS are superoxide dismutases (SODs), catalase (CAT), and glutathione peroxidase and reductase (GSH-Px) [18]. The nonenzymatic components including some small molecules such as vitamin A/C/E and glutathione act as cell structures or electron receptors against the damage from free radicals [19].

Mitochondria have been considered a major site of ROS production, where molecular O_2_ is reduced to O_2_^•−^ through complexes I and III by nicotinamide adenine dinucleotide/flavin adenine dinucleotide (NADH/FADH2). Monoamine oxidase, *α*-ketoglutarate dehydrogenase, and glycerol phosphatase dehydrogenase further contribute to generating O_2_^•−^ [20]. Mitochondrial dysfunction seems to be a common mediator triggering oxidative stress. Under conditions of normal mitochondrial homeostasis, a cell can eliminate physiological ROS and make metabolic adaptations. In NAFLD, however, increased mitochondrial fatty acid oxidation and tricarboxylic acid (TCA) cycle activity persistently supply reducing equivalents to the electron transport chain (ETC) [21]. This prolonged dysfunction in the respiratory complex promotes the generation of superoxide anion (O_2_^•-^). Notably, the uncoupling between *β*-oxidation, the TCA cycle, and ETC frequently results in inefficient lipid metabolism and ROS overproduction in the liver [22, 23]. In addition, the capability of mitochondria to reduce ROS levels is reduced in NAFLD, as indicated by decreased GSH metabolism [24], Mn superoxide dismutase (MnSOD) activity [25], and catalase activity [26]. Hence, either an increased production of prooxidant products or the dysfunction of the antioxidant system may induce oxidative stress. Accompanied by ROS accumulation, free fatty acid-induced hepatic lipotoxicity also promotes mitochondrial outer membrane permeabilization (MOMP) and alters the release of mitochondrial proteins and mitochondrial bioenergetics in NAFLD [27, 28].

Additionally, due to lack of histone protection, mitochondrial DNA (mtDNA) is highly sensitive to ROS. It is prone to damage and mutation, resulting in respiratory chain defects and decreased mitochondrial biogenesis under oxidative stress [29]. Oxidative damage to nuclear DNA impairs mitochondrial function and hinders the transcription of nuclear-encoded mitochondrial genes. For example, nuclear factor erythroid 2-related factor 2 (Nrf2), an essential modulator of antioxidant signaling that serves as a primary cellular defense against the cytotoxic effects of oxidative stress, has been reported to be decreased in NAFLD [30]. In the process of hepatic metabolism, specific polyunsaturated fatty acids (PUFAs) trigger lipid peroxidation, accompanied by increases in highly reactive aldehyde products such as malondialdehyde (MDA) and 4-hydroxy-2-non-enal (4-HNE) [31]. Thus, these mechanisms may eventually lead to a harmful cycle of mitochondrial damage and mitochondria-derived oxygen radicals.

In addition, the endoplasmic reticulum (ER) and peroxisomes are able to produce various kinds of ROS in liver tissues. Highly reactive molecules such as •OH, perhydroxyl radical (HO_2_•), H_2_O_2_, and ^1^O_2_ are produced from the reaction between O_2_•− and other molecules [32]. ER stress in the development of steatosis and subsequent generation of ROS aggravate the liver injury and promote the progression of NAFLD [33]. Moreover, excess of long-chain fatty acids (LCFAs) promotes the generation of H_2_O_2_ through increasing peroxisomal *β*-oxidation [34]. Similarly, very long-chain fatty acids (VLCFAs) enhance ROS production by cytochromes P4504A- and P4502E1-mediated microsomal oxidation [35]. In addition, several enzymes in the plasma membrane and cytosol are the producer of free radicals. For example, cytochrome P450 (CYP) enzymes play vital roles in the metabolism of drugs and other xenobiotics and regulate the generation of ROS and bioactivated intermediates [36]. Nicotinamide adenine dinucleotide phosphate (NADPH) oxidase (NOX), xanthine oxidase, cyclooxygenases, and lipoxygenases also act as important regulators in the reactions of xenobiotic metabolism [37].

It has been proposed that the gut microbiota acts as a vital role in developing NAFLD [38]. In patients with NAFLD, the abundance and composition of the microbiome are altered (dysbiosis) [39], accompanied by enhanced intestinal permeability [40]. As a result, bacterial lipopolysaccharides (LPS) are derived from the overgrowth of Gram-negative bacteria. Evidence has shown that the serum level of LPS increases 38–40% in patients with NAFLD compared with that in controls [41]. High liver exposure to LPS induces the excessive release of ROS due to impaired antioxidant system [42]. Moreover, in patients with NAFLD, endogenous ethanol also caused by some microbial species increases ROS formation in hepatic stellate cells (HSCs) and stimulates intestinal bacteria to release LPS [38]. Besides the role in increasing hepatic inflammation and oxidation, LPS acts on Kupffer cells (KCs) to upregulate cytokine receptors such as tumor necrosis factor-*α* (TNF-*α*) receptor, which may also be involved in ROS overgeneration [43].

**Figure 2:**
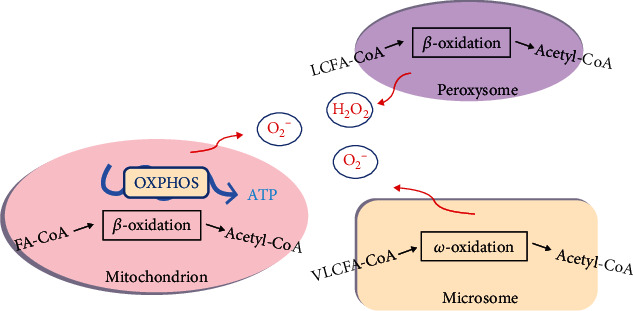
Main sources of ROS during the development of the fatty liver. In NAFLD, lipid oxidation induces the formation of reducing equivalents and causes an overflow of electrons through the mitochondrial respiratory chain (OXPHOS). Accumulation of long-chain fatty acids (LCFAs) increases peroxisomal *β*-oxidation, with consequent production of hydrogen peroxide. Excess of very long-chain fatty acids (VLCFAs) enhances microsomal oxidation with consequent generation of free radicals.

Overall, diverse sources of ROS and redox regulation may explain the pathogenesis of various liver diseases. In NAFLD, the increased formation of reducing equivalents results in an overflow of electrons from the mitochondrial respiratory chain, which induces higher ROS generation. ROS overproduction suppresses the capacity of antioxidant defense systems and causes further oxidative damage (Figure 2).

### 2.2. Implication of ROS and Oxidative Stress in the Development of NAFLD

Oxidative stress and imbalance of the redox state are distinctive characteristics of NAFLD [44]. Under physiological and pathological conditions, redox-dependent molecular alterations participate in the development of steatosis, providing new insights into the role of ROS as core regulators of liver lipid metabolism. Furthermore, increased ROS output and oxidative stress are identified as underlying mechanisms of insulin resistance, profibrogenic processes, and chronic inflammatory responses in NAFLD [10, 45]. This section outlines the recent knowledge on the regulators of ROS and oxidative stress in lipid metabolism and NAFLD progression.

#### 2.2.1. Redox Regulation of Crucial Enzyme Activity in Lipid Metabolism

The increased lipid uptake and synthesis and impaired lipid oxidation and removal lead to hepatic steatosis. The redox status modulates the activity of some key enzymes involved in hepatic lipid metabolism [10].

First, *de novo* lipogenesis (DNL) is activated when abundant glucose and insulin are in the plasma, usually in the postprandial state. However, under the selective insulin-resistant state in NAFLD, gluconeogenesis cannot be suppressed while DNL is promoted [46]. The human isotope-labeling studies showed that DNL is significantly elevated in patients with NAFLD, and the portion DNL accounts for intrahepatic triglyceride-palmitate increases as the severity of insulin resistance increases, about 11% in the lean group, 19% in the obese group, and 38% in the obese-NAFLD group [47, 48]. Saturated fatty acids (SFA) are the first product of DNL and can promote redox imbalance and the formation of reactive oxygen intermediates. In human HepG2 cells, SFAs were reported to increase ROS production by upregulating the expression levels of several components of the NADPH oxidase, including NOX3, NOX4, and p22phox [49]. Moreover, stearoyl-CoA desaturase -1 (SCD-1) can improve the toxic effects of SFAs [50]. While the downregulation of SCD-1 enhances delivery of FAs to mitochondria and oxidation in the fed state [51]. Once fatty acids reach the liver, they are bound to fatty acid-binding protein-1 (FABP-1) and then transport to the liver with the help of cell surface receptors such as fatty acid transport protein (FATP) family members and fatty acid translocase (CD36). In palmitic acid- (PA-) treated hepatocytes, H_2_O_2_ pretreatment abolished the effects of CD36 knockdown in attenuated oxidative stress [52]. Third, fatty acid oxidation usually takes place in mitochondria and peroxisome of energy-requiring tissues such as the liver and skeletal muscles. Hepatic *β*-oxidation mainly provides the fuel for hepatic basal energy requirements [53]. Liver-specific peroxisome proliferator-activated receptor *α* (PPAR*α*) knockout mice with impaired *β*-oxidation spontaneously are prone to NAFLD in aging even under a standard diet [54]. Notably, increased lipid oxidation and the TCA cycle are increased in NAFLD, indicating that hepatocytes enhance oxidation when counteracting lipid overload [55]. *β*-Oxidation is the primary producer to generate reducing equivalents (NADH or FADH2). The excess reducing equivalents cannot be resolved in the mitochondrial respiratory chain, resulting in higher ROS generation. Peroxisomal *β*-oxidation and microsomal oxidation also contribute to the redox unbalance in NAFLD [56]. These changes increase hepatic reduction degree, as indicated by alterations in the NADH/NAD^+^ ratio [57]. The increased ratio suppressed the activities of acyl-CoA dehydrogenase (LCAD) and *β*-hydroxyacyl-CoA dehydrogenase (*β*-HAD), which are involved in the pathway of fatty acid oxidation [58, 59]. Lastly, the export of lipids is another way for the liver to reduce lipid accumulation. Lipoproteins, such as chylomicrons (CM) and very-low-density lipoproteins (VLDL), contain core lipids like triglycerides and cholesterol esters. Hepatic endoplasmic reticulum synthesizes VLDL with apolipoprotein B (ApoB) and triglyceride with the help of microsomal triglyceride transfer protein (MTTP). This process enables the liver to alleviate endogenous triglycerides by secreting water-soluble VLDL into circulation [60]. However, a marked decline in VLDL secretion is observed in the insulin-resistant state of NAFLD. The unbalance between lipid droplets production with VLDL secretion leads to hepatic steatosis [61, 62].

In addition, the role of cholesterol metabolism in NAFLD is also an attractive topic. Cholesterol can further induce the alteration of cellular redox status and associates with the progression of liver damage [50]. Previous studies reported that 3-hydroxy-3-methylglutaryl-CoA reductase (HMG-CR), the rate-limiting enzyme in the cholesterol synthesis pathway, may be modulated by its thiol redox status and induced by hepatic ROS [63, 64]. More investigations are needed to elucidate the role of ROS in cholesterol metabolism.

#### 2.2.2. Oxidative Stress Involvement in NAFLD Progression

Simple steatosis may progress to NASH with apparent inflammation, advanced fibrosis, and cirrhosis [8]. In 1965, Comporti first reported that increased lipid peroxidation levels in carbon tetrachloride- (CCl_4_-) treated rats and described the production of ROS in hepatic injury. Then, in 1972, slater and colleagues hypothesized that ROS plays a causative role in the progression of liver damage [65]. In the context of NAFLD, impaired redox status and ROS accumulation are the origins of hepatic maladaptive responses to fat accumulation, thereby leading to hepatic metabolic impairment and NASH progression [10]. Moreover, oxidative stress-related oxidized phospholipids accumulate and induce mitochondrial dysfunction in hepatocytes [66]. The mitochondrial GSH depletion is also induced by cholesterol accumulation in the progression of NAFL to NASH [67]. Mitochondrial DNA (mtDNA), released from fatty liver-damaged hepatocytes, causes liver inflammation by toll-like receptor 9 (TRL9) activation [68]. Koliaki et al. reported that the mtDNA levels are decreased in patients with more advanced forms of NAFLD [69]. Thus, increased oxidative stress triggers hepatic stress pathways, and maintaining cellular redox homeostasis is a promising strategy for NASH therapy [70].

The hepatocytes are the primary cells affected by lipotoxicity-induced oxidative stress in the liver. However, nonparenchymal cells (NPCs), including HSCs, liver sinusoidal endothelial cells (LSECs), and KCs, are also involved in oxidative stress-induced liver damage [71]. As we know, HSCs are responsible for extracellular matrix (ECM) deposition in the development of liver fibrosis. Cytochrome P4502E1- (CYP2E1-) induced free radicals can activate the transdifferentiating of HSCs. On the contrary, antioxidants could prevent the effect of ROS on increasing collagen production [72]. The NOX1- and NOX2- deficient mice exhibited improved ROS production and hepatic fibrosis in CCl_4_ or bile duct ligation-treated models [73]. In addition, mice deficient in antioxidant cytoglobin (Cygb) are susceptible to oxidative stress, inflammation, and fibrosis under diethylnitrosamine (DEN) or a choline-deficient diet [74]. Specifically, LSECs govern the regenerative process initiation, but oxidative stress damages the typical phenotype of LSECs. Aberrant LSEC activation in chronic liver injury induces fibrosis [75, 76]. In addition, oxidative stress increases M1 polarization and promotes proinflammatory cytokines in Kupffer cells [77]. Therefore, it is intriguing to investigate oxidative stress-targeting, possibly even cell type-directed strategies for treating NASH progression.

During liver injury, oxidative stress induces the activation of redox-sensitive transcription factors, such as nuclear factor-*κ*B (NF-*κ*B) and activator protein-1 (AP-1), leading to an inflammatory response and the activation of cell death pathways in hepatocytes. In NAFLD, ROS regulates NF-kB activation by increasing the expression of proinflammatory cytokine TNF-*α* [78]. NF-*κ*B, a significant regulator of the inflammatory response, plays a vital role in regulating the transcription of genes involved in the establishment of the immune and inflammatory responses [79]. Reduced NF-*κ*B activity by antioxidants has been proposed as a therapeutic target in NASH due to its anti-inflammatory properties [80, 81]. Moreover, in the development of steatohepatitis, E2-related factor 2 (Nrf2) acts as a significant regulator of the redox balance and mediates anti-inflammatory and antiapoptotic effects of antioxidants [82]. The release and activation of Nrf2 increase the expression levels of the antioxidant genes in hepatocytes with ROS accumulation [83], while Nrf2-knockout mice treated with methionine- and choline-deficient (MCD) diet show exacerbation of liver inflammation and steatosis compared to control mice [84]. Evidence has shown that the dysfunctional Nrf2 in patients with NASH is tightly involved in the grade of inflammation, but not steatosis [85]. In addition, upregulated Nrf2 in senescent hepatocytes is related to the activation of cocultured HSCs. The Nrf2 agonist sulforaphane remarkably inhibits the effect of lipid accumulation-induced hepatocyte senescence on activation of HSCs by the Nrf2-antioxidant response element (ARE) pathway [86]. A new study reported that the dysfunction of redox homeostasis induces hepatocytes to be highly susceptible to proteasome-associated metabolic stress. In comparison, insufficient PPAR*γ*/Nrf2-driven antioxidative response is the main factor [87]. Moreover, the interaction between NF-*κ*B and Nrf2 is also a noticeable target for NAFLD progression. Evidence showed that NF-*κ*B p65 subunit represses the Nrf2/ARE system at transcriptional level by competitive interaction with the binding domain of CREB-binding protein (CBP) [88]. NF-*κ*B dissociates from inhibitor kappa B (I*κ*B) and then translocates to the nucleus. Nrf2 negatively controls the NF-*κ*B signaling pathway by multiple mechanisms, including inhibiting nuclear translocation of NF-*κ*B and blocking the degradation of I*κ*B-*α* [89].

Overall, oxidative stress plays a central role in the pathogenesis of various liver diseases. Modulation of the antioxidant response emerges as a promising direction to prevent NAFLD progression. Moreover, monitoring oxidative markers can recognize liver dysfunction and observe the response to pharmacological therapies.

## 3. Transcriptional Regulation of Lipid Metabolism by NRs in NAFLD

**Figure 3:**
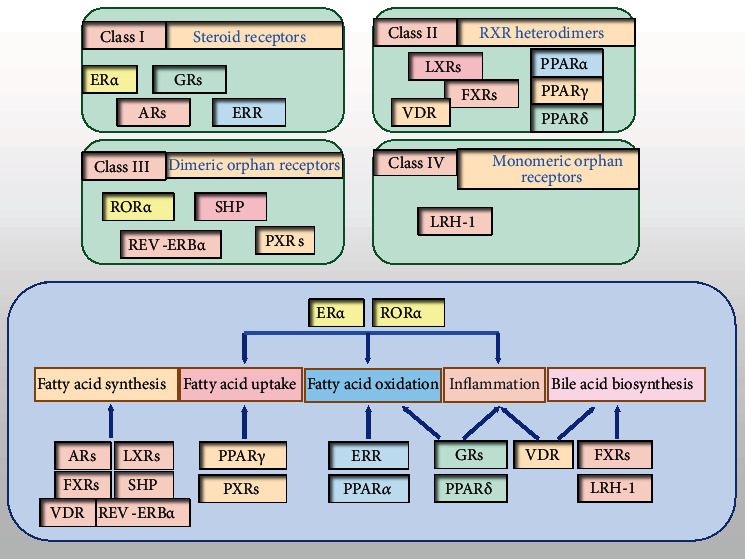
Nuclear receptor involved in hepatic lipid metabolism. Metabolic-related NRs can be classified into four classes according to their domains and ligands. Glucocorticoid receptors (GRs) coordinate energy requirements and mitochondrial oxidative phosphorylation enzyme biosynthesis, affecting lipid oxidation and the progression of inflammation. Androgen receptors (ARs), estrogen receptor *α* (ER*α*), and small heterodimer partner (SHP) contribute to the synthesis of fatty acids. ER*α* decreases fatty acid uptake and ROS generation. Fatty acid oxidation is favored by estrogen-related receptor (ERRs) and peroxisome proliferator-activated receptor *α* (PPAR*α*). PPAR*γ* regulates fatty acid uptake, and PPAR*δ* is a dual regulator of lipid utilization and inflammatory signaling. Pregnane X receptors (PXRs) play an essential role in lipid uptake by regulating the expression of CD36 and PPAR*γ*. Rev-erb*α*/*β* mainly modulates the activity of SREBPs to maintain lipid homeostasis, and it acts as a regulator in bile acid metabolism. Retinoic acid receptor-related orphan receptor *α* (ROR*α*) regulates lipid metabolism by modulating PPAR*γ*, AMPK, and liver-X-receptor *α* (LXR*α*) signaling. LXRs are vital for controlling lipid homeostasis by upregulating gene transcription involved in fatty acid and cholesterol metabolism. Vitamin D receptor (VDR) mainly acts as a regulator in lipogenesis and inflammation. Activation of VDR, farnesoid-X-receptor (FXR), and liver receptor homolog 1 (LRH-1) inhibit bile acid synthesis and prevent toxic accumulation.

Metabolic homeostasis is regulated through a network of programs, involving transcription factors, phosphatases, kinases, and NRs. NRs function directly on the genome to control gene transcription, often in response to small lipophilic ligands. Our group recently reported that nuclear receptor subfamily 2, group F, member 6 (NR2F6), acts as a causal factor in the development of NAFLD by binding directly to the CD36 promoter region in hepatocytes [90]. Moreover, several endogenous and exogenous lipids, including FAs and cholesterol, can serve as physiological NR ligands, and NRs also regulate the metabolism/catabolism of their respective ligands [91]. Notably, the cellular redox state may affect NR ligands or induce conformational changes in NRs to alter their DNA binding or nuclear import [91, 92]. The regulatory roles of some metabolic-related NRs in the development of NAFLD are specifically addressed below (Figure 3).

### 3.1. Introduction of NRs

NRs can be classified into four classes according to their domains and ligand: class I steroid receptors (e.g., glucocorticoid receptor (GR), androgen receptor (AR), estrogen receptor *α* (ER*α*), and vitamin D receptor (VDR)), class II retinoid X receptor (RXR) heterodimers (e.g., retinoic acid receptor (RAR), PPARs, liver-X-receptor (LXR), and farnesoid-X-receptor (FXR)), class III dimeric orphan receptors (e.g., pregnane X receptor **(**PXR), and class IV monomeric orphan receptors (e.g., liver receptor homolog 1 (LRH-1)). Class I classic nuclear receptors modulate lipid metabolism by reacting to traditional hormones including, but not limited to, thyroid hormone, glucocorticoids, estrogen, and testosterone. Class II nuclear receptors are linked to lipid metabolism and interact with metabolites as metabolic sensors. The third and fourth class of the nuclear receptor family is called orphan receptors, whose ligands have not been identified and functions remain elusive. A typical nuclear receptor has five regions in order: a variable N-terminal region (A/B) usually has a hormone-independent transactivation function, a conserved DNA binding domain (C) with two zinc-finger structures, a variable short hinge region (D), a conserved ligand binding-domain (E), and a variable C-terminal region (F). Regions C and E are signatures of nuclear receptors [93]. The human NR family can be classified into six evolutionary groups. In humans, all forty-eight NRs have these six domains except for 2 NRs in the subfamily NR0B lacking a DNA binding domain, but only half of the NRs are ligand-dependent. When ligands bind to these NRs, the ligand-binding domain (LBD) of the receptor changes conformationally to switch on the activity of the NRs. Notably, steroid receptors may modify enzymes and ion channels independent of transcriptional activation, namely, nongenomic effects [94]. Besides metabolic regulation through binding to multiple hormones, NRs also widely impact the embryonic development and maturation of several organ systems, signaling control in proliferation, and reproduction [95].

### 3.2. Metabolic-Related NRs

#### 3.2.1. Glucocorticoid Receptor (GR)

Chronic stress or excessive exposure to glucocorticoids (GCs) contributes to the pathogenesis of NAFLD [96]. GR mediates the action of GC and may act as a regulator on the effects of ROS in liver diseases. Mitochondrial GR coordinates the energy requirement with the mitochondrial oxidative phosphorylation enzyme biosynthesis, affecting the generation of free radicals [97]. In contrast, antioxidants can decrease the GR expression and increase the activity of the hypothalamus-pituitary-adrenal (HPA) axis in the pituitary [98]. Oversecretion of serum GCs induced by hyperactivity of HPA promotes ROS production in the brain tissues [99].

Lipid accumulation is a vital source of ROS production in the liver. Patients with Cushing's syndrome are inclined to develop hepatic steatosis [98]. GC receptors boost hepatic gluconeogenesis in response to oxidative stress and fasting. Long-term treatment with GCs usually leads to hyperglycemia and hepatic steatosis, partly because GCs can increase the expression of a set of circadian genes in the liver [100]. The detrimental metabolic actions of GCs can be mitigated by timed administration [101]. GR*β* coordinates with GR*α* in GC signaling, inducing high blood triglyceride levels and fatty liver in mice. The activity of glycogen synthase kinase 3 *β* (GSK3*β*) increases in the liver of GR*β*-Ad mice, in contrast to the decrease in PPAR*α* and fibroblast growth factor 21 (FGF21) [102]. GRs, binding to its ligand corticosteroids, recruit histone deacetylases 2 (HDAC2) and then translocase to the nucleus to bind GC response elements (GREs). The complex promotes the expression of anti-inflammatory proteins by reversing their histone acetylation [103]. Furthermore, GR-dependent fat mass- and obesity-associated (FTO) transactivation and m6A demethylation on mRNA of lipogenic genes are involved in the pathogenesis of NAFLD [104]. Importantly, the investigation of GR signaling provides new strategies for NAFLD treatment. E47 is required to activate GR target genes, as evidenced by free of GC-induced hyperglycemia or hepatic lipid accumulation in E47-knockout mice. Targeting E47 acts as a potential approach to improve the side effects of GC treatment because E47 can selectively regulate a subset of target genes [105]. In the liver, SET domain bifurcated 2 (SETDB2) serves as a GC-induced putative epigenetic modifier to regulate the GR-mediated gene activation. GR-SETDB2 dependent induction of insulin-induced gene 2 (Insig2) inhibits SREBP-1c-driven lipogenesis [106]. Dexamethasone-induced lipid accumulation can be reversed by hairy and enhancer of split 1 (Hes1) reconstitution and subsequent restoration of lipase gene expression (PNL and PNLRP2), highlighting the role of Hes1 in GR-mediated lipolysis. The deficiency of Hes1 in response to GC action explains the steatotic phenotype under starvation, myotonic dystrophy, and Cushing's syndrome [107]. Kruppel-like factor 9- (klf9-) mediated GR activation induces hepatic gluconeogenesis and hyperglycemia. Thus, targeting Klf9 might be a therapeutic approach to GC therapy-induced diabetes [108]. The increased expression of periostin in white adipose tissues mediates the effect of dexamethasone on hepatic lipid accumulation [109]. Moreover, other nuclear receptors also play roles in GR signaling. LXR *α*/*β* double-knockout (DKO) mice are protected from dexamethasone-induced insulin resistance by suppressing the key gluconeogenic enzyme phosphoenolpyruvate carboxykinase (PEPCK). While LXR*β* is required for the metabolic role of GR, it does not facilitate anti-inflammatory effects. The LXR*α*/*β* DKO mice hint at an opportunity to use selective GC agonists to induce anti-inflammatory effects without negative metabolic effects [110]. The selective GR modulator CORT118335 mimics the physiological GC action, stimulating the secretion of VLDL to delay the onset of NAFLD [111].

Notably, the tissue-specific action of GC gives it potential value in the metabolic modification of the liver, adipose tissue, and other tissues. 11*β*-Hydroxysteroid dehydrogenase type 1 (11*β*-HSD1) is an enzyme that promotes local GC regeneration. Mice with hepatic overexpression of 11*β*-HSD1 present increased hepatic lipid flux and impaired hepatic lipid clearance [112]. Global 11beta-HSD1 knockout mice show reduced expression of lipolytic enzymes (HSL and ATGL) in adipose tissue. Impaired hepatic 11*β*-HSD1 expression in ob/ob mice contributes to the pathogenesis of obesity [108]. Elevated in NAFLD but reduced in NASH, 11*β*-HSD1 has versatile roles in lipid metabolism and GC-related anti-inflammatory effects [113]. The 11*β*-HSD1 inhibitor RO5093151 slightly reduces liver-fat content in comparison with placebo [87]. Numerous compounds targeting 11*β*-HSD1 are under investigation, including natural products such as glycyrrhetinic acid and resveratrol, in the search for a therapeutic approach to NAFLD. However, unselective inhibition of 11*β*-HSD1 accelerates the activation of HSCs in the liver [108], suggesting that suitable target tissues should be established to bring into full play its inhibitory potency and low toxicity [114].

Overall, glucocorticoids modulate mitochondrial calcium homeostasis, ROS overproduction, and lipolysis [115, 116]. Multiple stressors activate the HPA-axis, which stimulates the adrenal secretion of glucocorticoids, thereby participating in the modulation of immune responses and inflammation [117]. These mechanisms may contribute to the effect of glucocorticoids in treating NAFLD/NASH. Targeting hepatic GR signaling by the star strand miR-192-3p is promising for treating fatty liver and insulin resistance [118]. However, given their complex pharmacology and effects on the immune system, more investigations are needed to evaluate the applicability of GRs as therapeutic targets in NAFLD.

#### 3.2.2. Androgen Receptor (AR) and Estrogen Receptor *α* (ER*α*)

The prevalence of NAFLD differs in gender and age [119]. Premenopausal women are less likely to develop NAFLD than men of the same age. In the same BMI level (27 ± 3 Kg/m^2^), postmenopausal women (60.2%) show a significantly higher prevalence of NAFLD than premenopausal women (42.9%), implying the protective effect of estrogen against hepatic steatosis [120]. Consistently, female mice receiving ovariectomy or tamoxifen treatment also suffer from TG accumulation [121].

Sex steroids are mainly inactivated in the liver. Both AR and ER are expressed in the human male and female livers. Independent of insulin resistance and obesity, sex steroids play vital roles in lipid and glucose metabolism by regulating the transcription of hepatic metabolic genes including carboxylase (ACC), transcription factor forkhead box protein O1 (Foxo1), SREBP-1, and FGF21. Androgen promotes the progression of hepatic fibrosis and HCC while estrogen has the countereffect [122]. Moreover, estradiol modulates mitochondrial metabolism and activities, including bioenergetics, oxygen consumption rate (OCR), and extracellular acidification (ECAR). Activation of nuclear respiratory factor-1 (NRF-1) transcription may mediate the effect of estradiol on mitochondrial function [123]. Loss of estrogen signaling contributes to hepatic oxidative damage induced by low levels of PGC-1*α*, exacerbating steatohepatitis in mice with high fat-diet [124]. ER*α*, the most well-characterized isoform of ER in the liver, can upregulate the expression of miR-125b to decrease fatty acid uptake and synthesis, which protects female mice from NAFLD [125]. Hepatic ER*α* genetic deletion/mutation mice develop severe hepatosteatosis regardless of gender [122]. Moreover, the estrogen-ER axis also plays a protective role in improving fatty acid oxidation and insulin response in adipose tissue and skeletal [123, 126]. Since cardiovascular events are more frequent in men and postmenopausal women, estrogen replacement therapy may be used in postmenopausal women to prevent cardio-metabolic consequences in NAFLD [127].

Estradiol has protective effects in males and females. Whereas androgen only reduces hepatic steatosis in the male group. Liver-targeted deletion of AR promotes fatty liver in male rodent models [128]. Since the incidence of obesity-related HCC is much higher in men than in women, androgen receptors may produce ontogenetic efficacy through alternative mechanisms, such as interaction with signal transducer and activator of transcription 3 (STAT3) [129]. AR plays a role in developing of neovascularization and liver cancer metastasis, which may participate in the progression from NASH to HCC [130].

The hepatic and whole-body metabolisms are improved in diabetic patients with estrogen treatment [48]. Consistently, hepatocyte ER*α* is considered a relevant molecular target for NAFLD prevention [131]. The effect of activation in ER signaling is complicated. At present, the clinical evidence for drugs that target ER is insufficient.

#### 3.2.3. Vitamin D Receptor (VDR)

VDR is highly expressed in gastrointestinal tract and endocrine tissues. Meanwhile, VDR is widely expressed in chronic liver disease patients' inflammatory cells and liver tissue [132, 133]. VDR mediates the genomic actions of vitamin D. It has been proposed that VDR may act as a druggable target for NAFLD in light of the discovery of vitamin D deficiency in NAFLD patients [134].

The primary active form of vitamin D is 1,25(OH)_2_D_3_, and the VDR ligand alters DNA-bound VDR homodimers into VDR-RXR heterodimers [135]. Exposing obese mice to 1,25(OH)_2_D_3_ prevents lipid accumulation and inflammation in developing NAFLD/NASH [136, 137]. However, vitamin D treatment has not consistently conferred expected therapeutic benefits. A new result of a meta-analysis indicated that vitamin D supplementation does not improve glucose metabolism parameters or lipid levels [138]. Moreover, plasma and hepatic ROS levels are decreased in the liver of VDR-deficiency mice compared to WT mice with acute hepatitis [139]. Several studies have shown that VDR-knockout mice are resistant to the development of liver steatosis and inflammation by decreasing lipid synthesis and promoting fatty acid oxidation [140]. In contrast, some long-term studies reported that VDR deficiency develops hepatic inflammation and fibrosis [141, 142]. Interestingly, nonparenchymal cells in the liver, including HSCs, KCs, and biliary epithelial cells, exhibit higher expression levels of VDR than in hepatocytes. Activation of VDR in hepatocytes promotes lipid accumulation [143], whereas inducing VDR in hepatic macrophages and HSCs attenuates hepatic inflammation and fibrosis [141, 142]. Moreover, ER stress induces increased VDR expression in hepatic macrophages. It has been proposed that VDR signaling regulates a shift between proinflammatory and anti-inflammatory activation during ER stress-induced inflammation to promote hepatic ER stress resolution [142]. Besides vitamin D, bile acids also act as ligands for VDR. It has been reported that hepatic VDR inhibits bile acid synthesis, thus preventing the liver injury in cholestasis [144].

Multiple genetic polymorphisms of the VDR gene or vitamin D-associated genes may explain these contradictory effects of vitamin D treatment in humans. Moreover, VDR-independent mechanisms or the binding ability of VDR to other endogenous ligands may play roles in mediating different effects of vitamin D. Thus, the diversity of VDR ligands and the cell type specificity of VDR activation would likely create difficulties in exploring VDR-targeted strategy for NASH treatment.

#### 3.2.4. Peroxisome Proliferator-Activated Receptors (PPARs)

PPARs are named for their interaction with peroxisome proliferators [145]. PPARs act as crucial regulators in lipid metabolism and determine synthesis rate of many enzymes involved in lipid, glucose, bile acid metabolism, adipocyte differentiation, and plasma apolipoprotein regulation. Three types of PPARs work in different organs [146].

PPAR*α* is mainly expressed in the liver and brown adipose tissue. It promotes energy utilization during fasting by boosting fatty acid oxidation and hepatic ketogenesis in the liver. PPAR*α*-knockout mice showed impaired fatty acid oxidation and a lower metabolic rate, resulting in hepatic steatosis, while the rate of VLDL secretion and gluconeogenesis remained unchanged [147]. PPAR*α* modulates liver-derived FGF21 in diabetic ketotic states [148]. Diet-induced obesity leads to disruption of circadian metabolic rhythms in PPAR*α* and SREBP-1. SREBP-1 regulates the production of endogenous PPAR*α* ligands to affect fatty acid oxidation [149]. Krüppel-like factor 6 (KLF6) [150], fatty acids [151], nutrition status [152], miR-27 [153], and other factors all affect the activity of PPAR*α*. Yoo et al. reported that fenofibrate, a PPAR*α* agonist, decreases hepatic fat accumulation through increasing TFEB-mediated lipophagy [154]. Moreover, PPAR*α* is also engaged in anti-inflammatory responses by interacting with NF-*κ*B and activator protein-1 (AP-1) [155]. PPAR*α* agonists reverse steatohepatitis and improve fibrosis [156]. In the livers of patients with NAFLD, increased poly (ADP-ribose) polymerase 1 (PARP1) activity represses PPAR*α* transactivation and may lead to weakened fatty acid oxidation [157]. PPAR*α* may act as a modulator in the antioxidant response, given the evidence that PPAR*α* expression is correlated with the Cu2^+^, Zn^2+^-superoxide dismutase (SOD) expression [158]. Moreover, the levels of PPAR*α* and its target genes including acyl-CoA oxidase type 1 (ACOX1) and carnitine palmitoyl transferases 1 (CPT-1) are decreased by H_2_O_2_ exposure in hepatocytes [159]. Clinical data showed that the expression levels of PPAR*α* negatively correlate with NASH severity [160].

PPAR*γ* regulates lipid storage and insulin sensitivity in adipose tissue, macrophages, and skeletal muscle. Hyperinsulinemia accelerates the development of hepatosteatosis in a PPAR*γ*-dependent manner [161]. PPAR*γ* protein expression is significantly downregulated in NAFLD, and PPAR*γ* transgene liver-knockout mice show a similar decrease in the expression levels of lipogenic genes such as fatty acid synthase (FAS) and SCD-1 [162, 163]. PPAR*γ* in macrophages of adipose tissue regulates genes involved in fatty acid synthesis, *β*-oxidation, and insulin-stimulated glucose uptake [149]. PPAR*γ* activators enhance cholesterol efflux in human macrophages [155] and suppress inflammatory cytokines in monocytes [164]. In fibrosis regulation, the reduced expression of PPAR*γ* results in inhibited HSC activation and increased collagen production [165]. In addition, PPAR*γ* agonists are insulin sensitizers that have been used to treat diabetes. Other studies have shown the prospects of nonagonist PPAR*γ* ligands for their antidiabetic actions [166]. Notably, a new study showed that PPAR*γ*2 translocates to the nucleus and activates signal transduction through a complex of PPAR*γ*2 and transportin 1 (Tnpo1) that forms via redox-sensitive disulfide bonds. The increased DNA-bound PPAR*γ* induces lipid accumulation in the liver. This evidence supports that a redox environment is a potential therapeutic target in the treatment of PPAR*γ*-related diseases [167].

PPAR*δ* is a dual regulator of lipid utilization and inflammatory signaling. Meanwhile, it can effectively improve insulin sensitivity and reduce atherogenic dyslipidemia [168]. PPAR*δ* stimulates FFA breakdown, fat depletion, and weight loss. microRNA-122 regulates hepatic fatty acid and cholesterol metabolism by targeting various genes, including PPAR*δ* [169]. The PPAR*δ* agonist GW501516 increased fat oxidation in skeletal muscle [170] and decreased serum ApoC-III concentration to help hepatic VLDL secretion in a small clinical data sample [171]. In a diet-fed obese diabetic mouse model, the PPAR*δ* agonist seladelpar (MBX-8025) reversed insulin insensitivity and improved NASH pathology independent of weight loss [172].

PPAR*β*/*δ* shares several similar functions to PPAR*α* in inducing fatty acid oxidation and improving NAFLD by functioning in the liver and other tissues [173]. Moreover, the effects of PPAR*β*/*δ* on NAFLD, including its capacity to decrease lipogenesis, improve inflammation and endoplasmic reticulum stress, alleviate insulin resistance, and attenuate liver injury [174]. PPAR*β*/*δ* agonists have been employed to prevent fibrosis in preclinical animal studies [175]. Thus, synthetic or natural ligand-induced activation of hepatic PPAR*β*/*δ* provides a promising therapeutic strategy for NAFLD. Despite no PPAR*β*/*δ* activator being approved for patients with NAFLD/NASH, various compounds are under clinical development at different stages.

#### 3.2.5. Liver X Receptor (LXR)

LXRs, including LXR*α* (NR1H3) and LXR*β* (NR1H2), are mainly expressed by the digestive tract where lipids are digested and absorbed. They are nuclear receptors that regulate the metabolism of several vital lipids, including cholesterol and bile acids [176]. LXRs upregulates cholesterol 7alpha-hydroxylase 1 (CYP7A1) in the reverse cholesterol pathway. Moreover, LXRs act as glucose sensors and strengthen fatty acid synthesis by activating SREBP-1c and carbohydrate responsive element-binding protein (ChREBP) [177]. As reported, hepatic insulin resistance leads to an increase in the activity of SREBP-1a, 1c and -2, resulting in elevated fatty acid synthesis [178, 179]. LXR*α* plays a crucial role in the insulin-induced proteolytic process to activate SREBP-1c. However, LXR agonists cannot affect SREBP-2 or its downstream targets [180]. Target genes of LXRs include hepatic cholesterol efflux modulator ATP-binding cassette transporters (ABCA1) and apolipoproteins as well as mitochondrial metabolic regulator PARP1 in brown adipose tissue and skeletal muscles [181]. LXR*α*/*β* also plays a role in the dynamic modulation of membrane phospholipid composition through Lpcat3, indirectly regulating the ER stress and inflammation in the liver [182]. LXR-null mice show impaired reverse cholesterol transport and increased atherosclerosis [183]. LXR*α*/*β*-deficient-ob/ob (LOKO) mice exhibits improved insulin sensitivity and weaken SREBP-1c and ChREBP activity in the liver accompanied by impaired hepatic lipogenesis [184]. Although liver-specific activation of LXRs does not impact reverse cholesterol transport, intestinal-specific LXR activation suppresses the absorption of cholesterol and improves lipoprotein profile [185]. When in the state of hypercholesterolemia, LXR*α* maintains peripheral cholesterol homeostasis [186], and LXR*β* can compensate for the antiatherosclerosis effect in the absence of LXR*α* [187]. Pharmacological activation of LXR by GW3965 and T0901317 increases transintestinal excretion of plasma cholesterol in different mouse models [185, 188]. Moreover, LXR functions are required for Kupffer cell identity and survival in response to NASH-induced environmental signals. These results show the regulator role of LXR in the development of NASH by controlling diversification in macrophage phenotypes [189].

The phosphorylation state of LXR*α* is associated with the progression of NAFLD [190]. LXR activity can be enhanced through deacetylation by sirtuin type 1 (SIRT1) [191]. Toll-like receptor- (TLR-) LXR signal crosstalk works under the regulation of transcription cofactor nuclear receptor coactivator 5 (NCOA5) [192]. Then, activation of adenosine monophosphate-activated protein kinase (AMPK) supports the S6 kinase 1- (S6K1-) mediated inhibition of LXR activity in lipogenic gene induction [193], while uncoordinated 51-like kinase 1 (ULK1) has the opposite function by reducing NOCR1 nuclear uptake and its interaction with LXR, which ends in a decrease in SCD-1 expression [194]. Fatty acid intake may also impact the expression of LXRs and its downstream targets ABCA1 and SREBP-1c [195].

However, some studies reported inconsistent experimental results about the role of LXR*α* in patients with NASH [196, 197]. Besides improving lipid accumulation in the liver, LXR*β*-selective and LXR*α*/*β*-dual antagonism may lead to hypercholesterolemia in nonhuman primates [198], which represents a barrier to the development of LXR antagonist as a therapy for NAFLD.

#### 3.2.6. Farnesoid X Receptor (FXR)

FXR is widely expressed in several tissues and has been demonstrated to be the primary sensor for modulating bile acids uptake and synthesis, gluconeogenesis, and fatty acid oxidation [199]. Gain of FXR function studies in nongastrointestinal tissues indicates that FXR signaling improves various experimentally induced metabolic and immune diseases [200].

Hepatic FXR expression can be upregulated by hyperglycemia and repressed by insulin. Our previous study showed that FXR downregulation accounts for the aging-induced fatty liver and ER stress represses FXR expression by inhibition of hepatocyte nuclear factor 1 alpha (HNF1*α*) transcriptional activity in old mice [201]. Moreover, we found that suppressing FXR expression by Yin Yang 1 (YY1) increases obesity-associated hepatosteatosis [202]. Interestingly, lean NAFLD patients have significantly higher FXR activity and a distinct microbiota profile, but their favorable metabolic profile not help resist hepatic lipid accumulation [203].

FXR agonists reduce lipogenesis by the interaction with LXR and small heterodimer partner (SHP) [204]. FXR-null mice show lower expression of SHP and higher serum and hepatic triglyceride levels [205]. FXR negatively regulates glycolysis and lipogenesis in the liver through inhibition of ChREBP [206]. FXR increases the expression and secretion of gene FGF21 [207]. Insulin sensitivity and glucose homeostasis are also impaired in mice with FXR depletion [208]. FXR-SHP-LRH1 pathway represses bile acid biosynthesis by targeting CYP7A1 [209]. Moreover, hepatic FXR mediates the protective effect of AMPK activators on oxidative injury and mitochondrial dysfunction induced by serum deprivation [210]. Intestinal reclamation of bile salts also works under the FXR-SHP-LRH1 pathway [211]. Interestingly, intestinal FXR takes charge of bile acid uptake [212] and changes hepatic lipidomics through the microbiome [213]. Hepatic FXR contributes to lipid accumulation under a cholesterol diet rather than intestinal FXR [214]. The pentose phosphate pathway regulates the expression of FXR in the liver, suggesting T2DM patients may suffer from lipid and bile acid dysregulation due to hyperglycemia [215].

FXR integrates the protein kinases A (PKA) and the forkhead box protein A2 signal in hepatic glucose production [216]. Src-mediated FXR phosphorylation after a meal maintains bile acid homeostasis [217]. The SUMOylation of FXR is higher in HSCs from NASH patients than healthy donors. Moreover, SUMOylation inhibitor can restore FXR activity, thus synergizing with FXR agonists when treating NASH [218]. FXR acetylation is regulated by SIRT1 and p300, which constitutively elevated in metabolic syndrome [219]. Besides the dysregulated acetyl/SUMO switch of FXR [220], the glucose-sensing O-GlcNAcylation pathway contributes to NAFLD in obesity [221].

Obeticholic acid, an FXR agonist, is approved by the FDA for biliary cholangitis therapy but not for NASH resolution. Nevertheless, FXR remains an attractive target for NAFLD/NASH. It is not clear whether redox states or ROS-derived compounds may directly regulate the FXR signaling pathway. This topic needs more investigation.

#### 3.2.7. Pregnane X Receptor (PXR)

PXR is abundantly expressed in the liver and gut, targeting metabolic enzymes and transcription factors such as CD36 and PPAR*γ* [222]. PXR ablation alleviates steatohepatitis in high-fat diet-induced obesity mice and genetic obesity model ob/ob mice, suggesting the therapeutic potential of PXR antagonists in NAFLD [223]. PXR target gene Cyp3a11 was consistently increased 3-4-fold in addition to the increased microsomal Cyp3a enzymatic activity at all stages of NAFLD [224]. Further, Di (2-ethylhexyl) phthalate- (DEHP-) induced ROS production activates the Nrf2 and nuclear xenobiotic receptor (NXR) system including aryl hydrocarbon receptor (AHR), PXR, and constitutive androstane receptor (CAR) in the development of liver injury [225]. Despite the promotion of hepatic steatosis and insulin resistance, PXR also shows antifibrotic and antiproliferative efficacy. Rifampicin activates PXR in human hepatic stellate cell line LX-2 and decreases the expression of fibrosis-related gene TGF-*β*1 and reduces the secretion of proinflammatory cytokine IL-6 [226].

It is worth noting that the consequence of PXR activation on overall metabolic health has not yet been fully elucidated, and varying experimental results on the effect of PXR activation or deficiency on metabolic disturbance have been reported [227]. Moreover, obese levels of parental mice decrease the hepatic expression of PXR in offspring [228]. At present, PXR is not being targeted in clinical trials for NAFLD therapy due to its uncertain role in hepatic metabolism.

#### 3.2.8. REV-ERB*α*/*β* and Retinoic Acid Receptor-Related Orphan Receptor *α* (ROR*α*)

Circadian rhythm, in other words, the sleep-wake cycle, regulates lipogenesis independent of the fasting-feeding process [229]. Circadian oscillations are observed in the expression of Rev-ERB*α*/*β* and ROR*α*/*β*/*γ* in the liver. REV-ERB*α*/*β* binds to RORE to recruit histone deacetylase 3 (HDAC3) and NCoR in rodent models to inhibit lipogenesis during daytime, while RORs bind to RORE instead of REV-ERB*α*/*β* at night [230].

REV-ERB*α* modulates the activity of SREBPs to maintain lipid homeostasis and regulates the expression of CYP7A1 to balance bile acid metabolism [231]. A large proportion of REV-ERB*α* target genes in hepatic lipid metabolism also requires the presence of HNF6 to work correctly [232]. Pharmacological activation of REV-ERB*α* by SR9009 attenuated hepatic steatosis, insulin resistance, inflammation, and fibrosis in mice with intestinal barrier dysfunction-related disorders and NASH [233]. Compared with REV-ERB*α* knockout mice, REV-ERB*α* and REV-ERB*β* double-knockout mice exhibit more severe hepatic steatosis, failing to recruit HDAC3 and NCoR in the liver, justifying the collaboration of REV-ERBs in hepatic lipid metabolism [234]. Hepatic REV-ERB*α* and Rev-ERB*β* double-knockout impairs daily rhythms of a subset of liver genes and alters the diurnal rhythm of de novo lipogenesis in mice. Moreover, the loss of hepatic REV-ERBs also remodels the rhythmic transcriptomes and metabolomes of nonhepatocytic cells within the liver [235]. In the light of the loop feedback in Clock/BMAL1 and REV-ERBs, both agonists and antagonists of REV-ERBs could be a potential therapeutic approach to reestablish metabolic balance [236].

In contrast to REV-ERBs, ROR*α* act as a transcriptional activator and coordinate the circadian rhythms of lipid metabolism and inflammation in the liver. ROR*α* recruit HDAC3 to PPAR*γ* promoters as a negative regulator of lipogenic genes [237]. Moreover, ROR*α* attenuates hepatic steatosis through AMPK activation and LXR*α* repression [238]. Liver-specific knockout of ROR*α* aggravates NASH development by impairing mitochondrial function. The expression level of PGC-*α* is positively related to ROR*α* in patients with NASH [239]. In mice models, ROR*α* decreases lipid peroxidation and inflammatory cytokine (TNF*α*, IL-1*β*) levels to prevent NASH. JC1-40, a ROR*α* activator, controls M2 polarization and reduces oxidative stress to improve symptoms of NASH [240, 241]. Targeting ROR*α* is an effective strategy for reducing ROS generation and increasing antioxidant capacity in endothelial cells and prepubertal cumulus cells [242, 243]. Moreover, ROR*α* regulates polarization in liver macrophages, which plays a fundamental role in liver fibrosis. ROR*α* agonist SR1078 validates that by suppressing HSC proliferation potently [244]. Whereas macrophage-specific knockout ROR*α* does not prevent insulin resistance and NASH [245]. Thus, the roles of ROR in different cell types need consideration. In addition, ROR*α* may increase its ligand maresin 1, which in return increases the expression and transcriptional activity of ROR*α*. This autoregulatory circuit provides a new potential therapeutic target for the NASH treatment [246].

In the liver of patients with NASH, ROR*α* expression is reduced [247]. The clinical application of targeting ROR*α* remains to be further investigated for NAFLD pharmacological therapeutics.

#### 3.2.9. Estrogen-Related Receptor (ERR)

ERR family is comprised of ERR*α*, ERR*β*, and ERR*γ*. Both in vitro and in vivo models, regulation of ERR*α* activity via genetic or pharmacological manipulation has been fundamental in delineating the vital roles of ERR*α* in lipid and carbohydrate metabolism, as well as in mitochondrial function under both physiological and pathological conditions [248]. The expression of fatty acid synthesis genes (Acly, Fasn, and Scd-1) shows a rise in ERR*α*-null mice, supporting the prominent role of ERR*α* in rapamycin-induced NAFLD [96]. Inhibition of ERR*α* decreases triglyceride biosynthesis and prevents hepatic steatosis. Targeting glycerophosphate acyltransferase 4 and glycerolipid synthesis is an important mechanism for ERR*α*-regulated NAFLD progression [249]. Moreover, ERR*α* participates in the weakened lipid oxidative catabolism after fasting-refeeding in mice [250].

In addition, liver-specific ablation of ERR*γ* normalizes blood glucose levels in db/db mice. GSK5182, an inverse agonist of ERR*γ*, may be a treatment option to inhibit hepatic gluconeogenesis [251]. ERR*γ* directly regulates the transcription of lipogenic gene srebp-1c via binding to an ERR-response element. Consistently, GSK5182 significantly improved NAFLD in chronically alcohol-fed mice by inhibiting SREBP-1c-mediated fat accumulation [252]. Moreover, the expression levels of ERR*γ* and fibrotic genes are elevated in liver tissue of obese patients. Overexpression of ERR*γ* increased fibrinogen expression in hepatocytes [253].

Given the experimental evidence, targeting hepatic ERR*α* activity may have therapeutic potential. The complex interplay of the three ERRs in the development of NAFLD and metabolic syndrome should be considered in future research and drug development.

#### 3.2.10. Small Heterodimer Partner (SHP)

In 1996, Seol and his colleagues reported that SHP is an orphan member of the NR superfamily that contains the dimerization and ligand-binding domain found in other family members. However, the conserved DNA binding domain is lacking in the SHP gene. In general, SHP is a negative regulator in receptor-dependent signaling pathways by inhibiting transactivation induced by the superfamily members with which it interacted [254]. In the liver, SHP involves the pathogenesis of steatosis by regulating the transcriptional activity of SREBP-1c [255]. SHP knockout mice show decreased expression of genes involved in lipogenesis (PPAR*γ* and ACC) and increased expression of genes involved in lipid oxidation and export (PPAR*α* and VLDL) [256]. A new study reported that SHP overexpression in mice inhibits lipogenesis in a DNA methyltransferase-3a- (DNMT3A-) dependent manner [257]. Moreover, SHP expression is regulated by other NRs in livers. Our previous study found that in obese mice, SHP deficiency blunted the effect of estrogen in improving hepatic steatosis [258]. FXR can bind to the SHP promotor region and induce its expression. FXR-SHP axis is closely associated with bile acid and lipid metabolism and represents a promising target for treating NAFLD. New evidence has shown that miR-802-mediated defective FXR-SHP regulation promotes insulin resistance and the development of fatty liver [204, 259]. However, the expression level of FXR, but not SHP, was decreased in the liver tissue of patients with NAFLD [260]. Notably, SHP may serve as a ROS-sensitive regulator in the effect of glycochenodeoxycholic acid (GCDCA) treatment on improving cell death and oxidation stress [261]. At present, the role of SHP in the diagnosis and treatment of NAFLD in humans remains unclear, and the data of related clinical trials are lacking.

#### 3.2.11. Liver Receptor Homolog-1 (LRH-1)

LRH-1 is expressed in the intestine, liver, pancreas, and ovary. In metabolic fields, LRH-1 regulates bile acid biosynthesis and reverses cholesterol transport [262]. SUMOylation, a kind of posttranslational modification, is primary for LRH-1 regulation. SUMO-deficient LRH-1 knock-in mice have better lipid metabolism and are less likely to develop atherosclerosis because of the inhibition of a set of genes linked to reverse cholesterol transport [263]. LRH-1 mutant mice have defects in SUMOylation and represent enhanced SREBP-1 expression and promoted DNL in high-fat diet or high sucrose diet [264]. Hepatic LRH-1 deficient mice show reduced hepatic glucose fluxes followed by a reduction in DNL because of the direct inhibition of glucokinase in transcription level by LRH-1, indicating LRH-1 plays a role in glucose-sensing in postprandial glucose and lipid metabolism [265]. Besides the glucose sensor, LRH-1 also functions as a phospholipid sensor to maintain the hepatic arachidonoyl phospholipids pool [266]. Coimmunoprecipitation confirms the synergy of FXR and LRH-1 in the activation of Cyp7A1 and fasn promoters in mice liver [267]. LRH-1 ligand dilauroyl phosphatidylcholine (DLPC) activates phosphatidylcholine signaling pathway and displays antidiabetic and lipotropic effects in mice [268]. LRH-1 agonist BL001 impedes *β* cell apoptosis in T2DM while it favors insulin secretion [266]. Notably, in the livers of LRH-1-knockout mice, the NADPH/NADP^+^ and GSH/GSSG ratios are decreased, supporting the role of LRH-1 in facilitating NADPH generation [269, 270]. In addition, evidence has shown that ROS production induced by a high concentration of palmitate in hepatocytes is reduced after LRH-1 agonist RJW101 intervention [271]. Thus, LRH-1 participates in metabolic processes to govern liver physiology and pathology. However, more clinical studies are needed to clarify the role of LRH-1 in treating NAFLD.

## 4. Clinical Research Findings Involving Metabolic Therapeutic Targets

We have briefly presented how NRs participate in modulating metabolic adaption and NAFLD/NASH progression. Given these findings, selecting transcription factors for the treatment of metabolic disorders is on the agenda. Here, we introduce the compounds ongoing in clinical trials.

### 4.1. PARs

PPAR*α* has been proposed as a promising therapeutic target based on its function in lipid and apolipoprotein regulation and inflammation and fibrosis resolution [272]. PPAR*α* agonist fibrates were introduced more than 35 years ago to improve the serum lipid profile and reverse atherogenic dyslipidemia [273]. In the obese animal models, fenofibrate treatment markedly improves hepatic oxidative stress and steatosis, ameliorates dyslipidemia, and improves insulin resistance [274, 275]. In addition, bezafibrate reduces plasma triglycerides (-49%) and hepatic triglycerides (-78%) in fructose-enriched diet- (FED-) treated rats [276]. However, the side effects of PPAR*α* agonist, including hepatomegaly and aminotransferase abnormalities, were observed in the animal studies need to be emphasized.

PPAR*γ* agonist pioglitazone belongs to thiazolidinediones. Thiazolidinediones improve insulin sensitivity by enhancing the differentiation of adipocytes. Pioglitazone 30 mg shows slight improvement in fibrosis in a 24 months clinical trial (NCT00063622)[277]. Thickened subcutaneous adipose tissue is frequently observed in thiazolidinediones, and pioglitazone is no exception. Heart failure, cardiogenic edema, and bone fractures in females [278] remain barriers for further clinical application.

PPAR*δ* agonist seladelpar decreases liver enzyme levels, inflammation marker levels, insulin resistance, circulating, and atherogenic dyslipidemia. It also reduces hepatic TGs [279]. However, it has been recently reported that seladelpar fail to decrease liver fat as quantified by magnetic resonance imaging in a phase 2 trial (NCT03551522).

Dual PPAR*α*/*δ* agonist elafibranor (GFT505) shows positive effects in glucose and lipid metabolism and reduces inflammation in NASH patients in a phase 2 clinical trial. Although elafibranor mildly increases serum creatinine, it is well-tolerated and does not exacerbate liver fibrosis [280]. A phase 3 clinical trial for patients with NASH is in progress (NCT02704403).

Dual PPAR*α*/*γ* agonist saroglitazar was first launched to treat diabetic dyslipidemia, uncontrolled by statins [281]. In NASH mice models, saroglitazar dose a better job than pioglitazone and fenofibrate in improving liver histopathology and biochemistry [282]. A phase 2 clinical trial (NCT03061721) of saroglitazar magnesium was finished in April 2020, aiming at lowering the serum ALT level in NASH. Current data showed that saroglitazar magnesium also improves the histological appearance in NASH. The drug firm Zydus Cadila has filed a new drug application of saroglitazar magnesium in NASH.

Pan PPAR*α*/*δ*/*γ* agonist lanifibranor shows positive effects on histology with a significant benefit over placebo for resolution of steatohepatitis, regression of fibrosis, and the combination of both [283]. A phase 2 clinical trial for patients with T2DM and NAFLD is in progress (NCT03459079).

### 4.2. FXR

FXR plays a critical role in maintaining bile acid and cholesterol homeostasis and regulating hepatic glycogen synthesis. FXR is a promising target for NAFLD/NASH [284]. FXR agonists targeting the gut-liver axis are promising for NAFLD/NASH for they not only relieve hepatic steatosis but also resolve fibrosis at histology level by antagonizing NF*κ*B [285, 286].

FXR agonists GW4064, GSK2324, chenodeoxycholic acid (CDCA), and fexaramine (Fex) have been tested in rodent models. GW4064, a synthetic agonist of FXR, lowers blood glucose and improves hepatic glycogen storage in normal and db/db mice regardless of whether they are fasted or fed [287]. GW4064 suppresses hepatic apolipoprotein CIII and apolipoprotein A-I [288] expression to prevent mice from coronary heart disease. FXR activation with the FXR agonist GSK2324 controls hepatic lipids via reduced absorption and selective decreases in fatty acid synthesis. The results in tissue-specific FXR KO mice show that hepatic FXR controls lipogenic genes, whereas intestinal FXR controls lipid absorption [289]. FXR activation by chenodeoxycholic acid (CDCA) in Zucker (fa/fa) obese rats reverse insulin resistance and hepatic steatosis [290]. Intestine-selective FXR inhibition by glycine-*β*-muricholic acid (Gly-MCA) improves metabolic dysfunction by reducing intestinal-derived ceramides [291]. Gut-restricted FXR agonist fexaramine (Fex) induces browning white adipose tissue, increases the metabolic rate in brown adipose tissue, alters bile acid composition, and improves hepatic steatosis and insulin sensitivity [213]. Fex improves FXR-gut microbiota-TGR5-GLP-1 signaling and increases FGF15 secretion without changing appetite in mice [292].

FXR agonist obeticholic acid successfully lowers serum markers representing hepatocellular injury (ALT, AST) and oxidative stress (GGT) in mice. Obeticholic acid also lowers serum LDL-C and increases liver LDLR expression [293]. In human patients, obeticholic acid (trade name Ocaliva) was first approved to treat primary biliary cholangitis for its function in reducing alkaline phosphatase and bilirubin levels to prevent cirrhosis [294]. Besides the anticholestatic and antifibrotic effects, obeticholic acid shows great potential in treating NAFLD. Obeticholic acid shows efficacy in improving the insulin sensitivity of NAFLD and T2DM patients. However, it also causes an increase in LDL and a reduction in HDL [162]. Biopsy proved the histologic improvement by obeticholic acid in parallel to the change of aminotransferases [295]. In the interim analysis from a phase 3 clinical trial, obeticholic acid 25 mg daily significantly improved histological endpoints in advanced fibrosis due to NASH compared to the 10 mg low dose group or placebo [296]. Side effects like pruritus can be conquered by symptomatic treatment, and elevated LDL cholesterol levels can be treated with lipid-lowering agents like statins. Obeticholic acid is the first drug application for NASH-related liver fibrosis accepted by the FDA.

Nonsteroidal FXR agonist cilofexor (GS-9674) (NCT02854605) [295], nonbile acid FXR agonist tropifexor (LJN452) (NCT02855164) [297], and nidufexor (LMB763) (NCT02913105) [298] are undergoing phase 2 clinical trial in NASH patients.

## 5. Hormones Affecting the Expression of NRs in the Hepatic Lipid Metabolism

### 5.1. Thyroid Hormones

Thyroid hormones 3,5,3′-triiodothyronine (T3) and 3,5,3′,5′-tetraiodothyronine (T4) play essential roles in developmental process, differentiation, growth, and metabolism in cells through the genomic or nongenomic pathways. The genomic action occurs through their interaction with nuclear receptors TR*α* and TR*β*, together with coactivators or corepressors to modulate gene expression and protein synthesis [299]. Thyroid hormones are potent regulators in body weight, lipogenesis, lipid metabolism, and insulin resistance. Evidence confirmed that the liver is a significant target for thyroid hormones [300]. Moreover, TR*β* is mainly expressed in the liver tissue, and TR*α* is more common in bone and cardiovascular organs. Mice with a dominant-negative mutation in TR*β* (Thr*β*^PV/PV^) develop hepatic steatosis and have larger livers. Moreover, these mutated mice exhibit upregulated activation of PPAR*γ* signaling and reduced fatty acid *β*-oxidation, leading to the development of steatosis [301]. In addition, thyroid hormones also regulate the expression and activities of many NRs involved in lipogenesis, such as LXR [222]. HMG-CoA reductase, the limiting enzyme of cholesterol synthesis, is inhibited by thyroid hormones [302]. Meanwhile, liver fibrosis begins with injury and mitochondrial dysfunction in cells. The increased free fatty acids and ROS induce lipid peroxidation and activate HSCs. Under liver injury, the dominant hormone receptor becomes TR*α* instead of TR*β*. TR*α* produces a more robust wound-healing response in the fibrogenic process [303].

### 5.2. Melatonin

The pineal hormone melatonin is synthesized from tryptophan via 5-hydroxytryptamine and is considered a potent regulator of oxidative damage in different vertebrates [304]. Melatonin acts through specific receptors, including melatonin 1 (MT1), MT (2), and MT (3) receptors as well as a nuclear receptor belonging to the orphan nuclear receptor family. M1 is the one mainly expressed in the liver tissue. Exciting, therapeutic effects of melatonin on improving fatty liver are observed in obese rats by inhibiting oxidative damage [305]. Moreover, in diabetes and obesity, melatonin supplementation has been found to protect liver function by recovering mitophagy via blockade of nuclear receptor 4 A1 (NR4A1) [108]. In hepatocytes exposed to H_2_O_2_, melatonin treatment reduces the levels of oxidative stress and ROS generation, thereby improving liver damage [306]. Meanwhile, melatonin induced a dose- and time-dependent inhibition on the proliferation of hepatocytes [307]. Chronic CCl_4_ exposure induces collagen deposition and oxidative stress, while melatonin protects against liver fibrosis via increased mitophagy and mitochondrial biogenesis [308]. Therefore, melatonin is considered a potent antioxidant drug to improve fatty liver [309].

## 6. Summary and Outlook

The onset of NAFLD is characterized by changes in redox status in the hepatocellular system that lead to ROS generation and impaired hepatic metabolism. Oxidative stress is also a causative factor in the pathologies of the fatty liver. The molecular mechanisms accounting for these alterations are not entirely understood, but activation of NRs plays a vital role in regulating the redox status and the metabolic network. Antioxidant molecules favorably modulating the cellular redox environment may also regulate NRs that play a role in lipid metabolism. This autoregulatory circuit provides more potential therapeutic strategies for NAFLD/NASH treatment.

Nuclear receptors have largely maintained their dominance of the drug target space for human use [310]. Based on the vital role of NRs in regulating hepatic metabolism and on the promising results observed in animal models with NAFLD, drugs which interfere with NRs are among the strongest candidates for NAFLD therapy. However, several clinical trials utilizing pharmacological manipulation of NRs have yielded conflicting results about the efficacy and safety of these drugs. Despite specific favorable metabolic effects, PPAR*α* activator fibrates have failed to improve hepatic steatosis or NASH in humans [311, 312]. PPAR*γ* agonist rosiglitazone has shown the impact of resolution on hepatic steatosis but not on NASH. It might increase in bone fractures, fluid retention, and cardiac decompensation [313–315]. In practice, considering drug safety, pioglitazone is the only thiazolidinedione (TZD) in use clinically today for the treatment of T2DM. Although previous studies have indicated a limited efficacy of activating individual PPARs, ongoing clinical trials show that dual and pan-PPAR agonists might serve as promising strategies for NASH therapy. Moreover, the FXR agonist obeticholic acid shows significant benefit in phase 3 interim results and remains the candidate for first conditional approval as a NASH therapeutic [316]. Additional research is needed to confirm this promise and address concerns about tolerability and side effects.

Drug discovery programs targeting NRs have been greatly facilitated by the emergence of ligand-binding domains and the resulting opportunities to identify new chemical activators/inhibitors. NRs act directly on the genome to control transcription. Unlike targeting traditional drugs, targeting transcription factors and their cofactors results in less drug resistance but is more likely to have other side effects. For instance, PPAR*γ* agonist rosiglitazone treatment-induced adverse events such as bladder cancer and heart failure have become highly aware in clinical application. Thus, selective inhibition/activation of a transcription factor may require a low dose with minor side effects. Moreover, it seems that the next-generation dual-PPAR or pan-PPAR agonists are presently the most promising strategies, addressing the therapeutic benefits of targeting more than one PPAR subtype in the treatment of NASH [146]. In addition, given the diverse actions of NRs in multiple organs and how they affect metabolic crosstalk with various layers of complexity, clarifying the tissue-specific and cell-type-specific roles of NRs is essential for precise pharmacological treatments. Nowadays, the advancements made to the development of “human-on-a-chip” models seem as effective strategies for testing novel drug candidates. The system provides a simple but unique platform to evaluate preclinical drug efficacy and reassess human dosing regimens [317, 318]. Moreover, by implementing in these chips, patient-derived stem cells carrying high-risk genetic backgrounds for developing NASH, the evaluation of personalized therapies might ever become a reality [319]. Furthermore, some natural compounds have been reported to treat NAFLD by acting on NR-targeted pathways with fewer adverse reactions, presenting a promising therapeutic prospect [320]. These drugs are naturally present in the human body and function by stimulating the physiological status. Notably, in addition to hepatocytes, targeting NRs should include anti-inflammation/fibrosis in nonparenchymal cells. NR ligand-based therapies are not the only strategy for NAFLD. Targeting posttranslational modifications such as acetylation of NRs and coregulators is also a promising direction for dealing with changes in the redox-microenvironment. Therefore, targeted redox-dysregulated NRs is a promising strategy for treating NAFLD.
